# Learning and generalizing non-adjacent dependencies in 18-month-olds: A mechanism for language acquisition?

**DOI:** 10.1371/journal.pone.0204481

**Published:** 2018-10-11

**Authors:** Ileana Grama, Frank Wijnen

**Affiliations:** 1 Department of Humanities, Utrecht University, Utrecht, The Netherlands; 2 Utrecht Institute of Linguistics OTS, Utrecht, The Netherlands; Universitat de Barcelona, SPAIN

## Abstract

The ability to track non-adjacent dependencies (the relationship between a_i_ and b_i_ in an a_i_Xb_i_ string) has been hypothesized to support detection of morpho-syntactic dependencies in natural languages (‘The princess is reluctantly kissing the frog’). But tracking such dependencies in natural languages entails being able to generalize dependencies to novel contexts (‘The general is angrily berating his troops’), and also tracking co-occurrence patterns between functional morphemes like is and ing (a class of elements that often lack perceptual salience). We use the Headturn Preference Procedure to investigate (i) whether infants are capable of generalizing dependencies to novel contexts, and (ii) whether they can track dependencies between perceptually non-salient elements in an artificial grammar aXb. Results suggest that 18-month-olds extract abstract knowledge of a_b dependencies between non-salient a and b elements and use this knowledge to subsequently re-familiarize themselves with specific a_i__b_i_ combinations. However, they show no evidence of generalizing a_i__b_i_ dependencies to novel a_i_Yb_i_ strings.

## Introduction

The remarkable ability of children to acquire their native language within a few years has often been attributed, at least partially, to their ability to detect distributional properties of the input. Distributional learning mechanisms have been successfully studied in both adult and infant populations. Thus, both adults [1] and infants [2] segment words from the input based on transitional probabilities between syllables, and use distributional cues to infer phonetic categories [3–4]. Both learn simple grammars and generalize those grammar rules to novel stimuli [5–6] and use frequent frames in the input (*to___it*) to infer lexical categories (*to V it*) [7–8]. Finally, both adults and infants detect the adjacent dependencies that determine phrase structure [9], and the non-adjacent dependencies that indicate more remote syntactic relationships [10–11]. The current study is concerned with the learning ability that allows both adults and infants to identify non-adjacent dependencies (NADs): co-occurrence patterns between elements that are non-adjacent in a sequence.

Gómez [10] showed that when exposed to a simple language composed of *aXb* strings (e.g. *tep kicey rud*, presented in a sequence, separated by pauses), both adults and 18-month-old infants learned the specific dependencies between non-adjacent *a* and b elements (e.g. between *tep* and *rud*), but only when the set of intervening bisyllabic *X*s was sufficiently large. When faced with a grammaticality judgment task, subjects showed a significant preference for consistent *a*_*i*_*Xb*_*i*_ over inconsistent *a_*i*_*Xb*_*j*_ dependencies when *X* could be instantiated by 24 different nonce words, but not 12. (Note: in this paper we use indexation to identify specific elements in a class: thus, *a*_*i*_ is a specific nonce-word from the class of *a* words, which in a dependency predicts the occurrence of a specific word *b*_*i*_ from the class of *b* words, but NOT of the word *b*_*j*_; the *i* and *j* indexes serve to indicate whether *a* and *b* are part of the same dependency *a*_*i*_*_b*_*i*_ or different dependencies *a_*i*_*_b*_*j*_). This suggested that the high variability of elements spanned by a dependency is a distributional cue that facilitates NAD-learning.

Gómez and Maye [11] confirmed the importance of **variability** in the *X*-element for learning *a*_*i*_*_b*_*i*_ dependencies, and studied the developmental timeline of NAD-learning in infants. Children exposed to a simple *a*_*i*_*Xb*_*i*_ language subsequently showed a significant discrimination between ‘correct’ *a*_*i*_*_b*_*i*_ and ‘incorrect’ *a*_*i*_*_b*_*j*_ dependencies at 15 and 18 months, but not at 12 months. Similarly, Santelmann & Juszcyk [12] showed that 18-month-olds but not 15-month-olds could track non-adjacent morpho-syntactic dependencies in natural languages (e.g. ‘The archaeologist *is* digg*ing* for treasures’ vs. ‘*The archaeologist *can* digg*ing* for treasures’). This NAD-learning ability, which arguably develops around the age of 15 months, narrowly precedes and may, perhaps, support the acquisition of NADs in natural languages around the age of 18 months.

Newport & Aslin [13] showed that, in adults, NAD-learning is modulated not only by distributional cues but also by **perceptual cues**. They tested learning of dependencies in continuous strings (i.e. without separating the *aXb* sequences by pauses like Gómez [10]), either between vowels over consonants, or consonants over vowels, or between syllables over syllables. Participants could learn segmental dependencies, but were unable to show a preference for consistent (*a*_*i*_*Xb*_*i*_) over inconsistent (*a_*i*_*Xb*_*j*_) strings in the test phase when dependencies were instantiated between syllables. The authors proposed that this pattern of results could be explained by Gestalt principles of perception guiding the learner to discover dependencies between dependent elements perceptually similar to each other, and distinct from the intervening material. They suggested that dependencies in natural languages may be specifically adapted to this learning constraint: for instance verb stems in Semitic languages are consonantal templates (*k-t-b* ‘to write’) where the vowels inserted in between mark tense, voice etc. (e.g. *katab* ‘wrote’). Thus, the NAD-learning exhibited in their study could help learners identify the verb stem and the verb morphology by tracking dependencies between consonants and between vowels separately.

Grama, Kerkhoff & Wijnen [14] extended Newport & Aslin’s [13] discussion of the relevance of NAD-learning to language acquisition. They showed that adult learners were capable of **generalization** of *a*_*i*_*Xb*_*i*_ dependencies to novel contexts (*a*_*i*_*Yb*_*i*_), where the intervening element *Y* was withheld from familiarization. This task, it was argued, more naturalistically approximated the task that infants may be faced with in language acquisition, infants are exposed to morpho-syntactic dependencies in a limited number of context (e.g. *The princess* ***is*** *gently kiss****ing*** *the frog*) but must acquire productive morphological rules that apply in novel contexts (e.g. *The general* ***is*** *angrily berat****ing*** *his troops*), independent of the intervening material. Previous studies have shown that infants are capable of generalizing the patterns they learn to novel contexts from a very young age [6,15–16] but, to our knowledge, none so far have tested this generalization ability with respect to NAD-learning.

Furthermore, Grama et al. [14] also picked up on the proposal that Gestalt principles guide NAD-learning and extended it to the domain of prosody. This study built on the observation that in many languages, morpho-syntactic dependencies are instantiated between functional morphemes, or functors (frequent morphemes, like auxiliary verbs, determiners–like ‘the’ and ‘a’–inflectional morphemes like plural ‘-s’, past tense ‘-ed’, etc., which have an important grammatical function but no substantive semantic content, unlike lexical morphemes such as verbs, nouns, etc.). These are often perceptually/prosodically reduced: they are shorter in duration, with lower relative amplitude, and simpler syllabic structure, and often have the status of prosodic clitics [17–20]. In an *a*_*i*_*Xb*_*i*_ language, the properties of the *a*_*i*_ and *b*_*i*_ elements were manipulated to be, in turn, (i) more, (ii) equally, or (iii) less perceptually salient (in terms of pitch, duration, etc.) than the *X* elements. Results showed that adults were able to detect the *a*_*i*_*_b*_*i*_ dependencies only when the dependent elements were either (i) more, or (iii) less, but not (ii) equally prosodically prominent compared to the *X*, and only when the prosodic status of the *a*_*i*_*/b*_*i*_ elements was consistently marked by both perceptual/acoustic cues (pitch, duration) and pause cues.

The study concluded that the adult learners were integrating pause cues and the perceptual properties of the *a*_*i*_*/b*_*i*_ elements into abstract representations of their prosodic prominence relative to the *X* elements. This was suggested to corroborate Newport & Aslin’s [13] account in terms of Gestalt principles of perception: learners were marking the elements in terms of their prosodic status and were guided to detect dependencies strictly between elements that were similar to each other but distinct from the intervening material. The evidence provided by this study suggests that natural languages may provide useful perceptual cues to the detection of morpho-syntactic dependencies. Whereas adults were shown to exploit these cues in NAD-learning, no study to date has confirmed whether infants too are capable of using the same cues to detect non-adjacent dependency structures.

Briefly put, both adult and infant learners are capable of tracking dependencies between non-adjacent elements in spoken input, and this NAD-learning ability has been hypothesized to support the acquisition of morpho-syntactic dependencies in natural languages. While adults have been shown to track dependencies between both highly salient *and* non-salient elements in the input, and to generalize these dependencies to novel contexts, there is little evidence that infants possess the same abilities. However, in order to employ NAD-learning as a mechanism for the detection of morpho-syntactic dependencies in natural languages, children must detect dependencies between often low-salience functional morphemes and generalize these dependencies to novel contexts.

In the current study we set out to investigate the hypothesis that NAD-learning in infants can, in principle, support the detection of morpho-syntactic dependencies in natural languages. We test two separate research questions: (i) are infants able to generalize the dependencies they detect to novel contexts?, and (ii) are they capable of detecting NADs between prosodically less salient elements over prosodically more salient elements?

Experiment 1 tests whether 18-month-old infants can generalize NADs by detecting previously learned dependencies in novel strings, with unfamiliar intervening elements. Experiment 2 investigates whether 18-month-olds are capable of detecting dependencies between prosodically reduced elements in the same way that adults are, and whether Gestalt principles of perception guide learning in infant learners, not just adults.

## Experiment 1

### Introduction

This experiment investigates the ability to generalize patterns between non-adjacent words to novel contexts. This ability seems crucial for a learning mechanism that may support the acquisition of morpho-syntactic dependencies as in ‘*The princess* *is* *lovingly kiss**ing* *the frog’*, since these morpho-syntactic rules must apply irrespective of the sentential context in which they occur (‘*The king* *is* *angrily glar**ing* *at the prime-minister’*).

Infants as young as 7 months have been shown to derive abstract representations of same/different patterns in short strings and generalize them to novel stimuli. Marcus et al. [15] showed that 7-month-olds familiarized with trisyllabic strings with the structure ABB (e.g. *ga ti ti*, *li na na*) showed a subsequent preference for novel ABB (e.g. *wo fe fe*) versus ABA (e.g. *wo fe wo*) strings at test, even though they had never heard either of them before. Gómez & Gerken [6] also exposed 11–12 month-olds to a finite-state grammar, where sensitivity to adjacent probabilities was crucial to detecting the structure of the language. Infants familiarized with a set of strings generated by that grammar (e.g. *fim jed tup fim jed tup*) subsequently showed a preference for completely novel strings that were consistent with the grammar (e.g. *pel rud jic pel rud jic*) over novel strings that weren’t (e.g. *pel tam pel pel pel jic*). These findings indicate an early ability to develop abstract representations of structure that are not linked to specific stimuli, but can be applied to different sets of stimuli. However, these studies investigate generalization of repetition-based patterns which are highly salient and easy to detect by even the youngest learners (see [21] and references therein for a discussion of how repetition-based learning may rely on different attentional mechanisms, that develop earlier than NAD-learning). Although we cannot discern whether infants detect ABA patterns using the same mechanism that allows them to track *aXb* dependencies, it should be noted that while ABA requires infants to generalize across both A and B novel elements, NAD-generalization requires maintaining item-specific representations of the *a* and *b* items and their one-to-one mappings, and generalizing only across the intervening *X* element.

To our knowledge no published studies so far have specifically investigated infants’ ability to generalize one-to-one dependencies between non-adjacent elements to novel intervening elements. Lany & Gómez [22] showed that 12-month-olds infants acquiring *aX* adjacent dependencies (between a grammatical marker *a* and a category *X*) can subsequently generalize these to non-adjacent *acX* dependencies in which a novel *c* is inserted into the dependency. This suggests that infants do not simply detect dependencies but are also capable of generalizing those dependencies to novel contexts, at least when the dependency is instantiated between a word and a word-category.

We expose infants of 18 months to an artificial *aXb* language and test their acquisition of the *a_b* dependency (between specific words) with novel *aYb* strings. Based on the findings above, we predict that infants with a mature ability to detect NADs in an unfamiliar language (i.e. most infants at the age of 18 months) should also be able to generalize those dependencies to novel contexts. We employ a similar methodology as used in previous NAD-learning studies with infants [10–11,23], but only slightly adjust the test phase to address the research question.

### Materials

Subjects were exposed to a language *aXb* similar to Gómez’s [10], with stimuli adapted to Dutch taken from Kerkhoff et al. [23], a study that replicated the findings of Gómez & Maye [11] with Dutch 18-month-old infants (with and without a genetic risk for dyslexia). The language contained 2 *a*_*i*_*_b*_*i*_ dependencies (*tep/sot__lut/jik*) and 24 bisyllabic intervening *X*s (*wadim*, *kasi*, *poemer*, *kengel*, *domo*, *loga*, *gopem*, *naspu*, *hiftam*, *dieta*, *vami*, *snigger*, *rogges*, *densim*, *fidang*, *rajee*, *seta*, *noeba*, *plizet*, *banip*, *movig*, *sulep*, *nilbo* and *wiffel*), combined exhaustively into a total of 48 familiarization strings. The *X* set size of 24 is the largest set size used previously in this type of experiment [10–11,23] and should therefore be optimal not just for prompting NAD-learning but also for inducing generalization over the intervening *X* class.

Two Grammar Versions were created (G1, G2), which differed only in the *a*_*i*_*_b*_*i*_ pairings (G1 had the dependencies *tep_lut* and *sot_jik*, whereas G2 had the dependencies *tep_jik* and *sot_lut*), such that the strings that were grammatical to G1 were ungrammatical to G2 and vice-versa. Infants were randomly assigned to one or the other Grammar Version, to control for potential stimulus biases. Because we wanted to investigate children’s ability to generalize the dependencies learned to novel contexts, we created test strings employing three *novel X*-like elements we will call *Y*s (*klepin*, *lotup*, *tarsin*). Each of the three *Y*s was combined with the dependencies of G1 and of G2, to create novel test strings that were either consistent with G1 or with G2.

The stimuli were recorded in a sound attenuated booth, at 48kHz, using a TASCAM DA-40 DAT-recorder. A female speaker read out *aXb* strings in a child-friendly voice, laying special emphasis on the *a/b* elements. The string-initial *a* and string-final *b* tokens were spliced from these recordings, in replication of Grama et al. [14]. Also, in order to replicate the properties of the stimuli in those experiments (and compare our findings with infants with the previous findings with adults), we recorded the *X*s/*Y*s separately, in carrier sentences in Dutch where they occupied the slot of the direct object noun:

(1) *Ik zie de ____ in de tuin*.
  I see the __ in the garden.
  e.g. *Ik zie de wadim in de tuin*.

Acoustic properties of the *a*, *b*, *X* and *Y* tokens, as analyzed using Praat 5303, are presented in S1 Table, alongside the acoustic properties of tokens employed in Kerkhoff et al. [23]. As can be seen, in both sets of stimuli the *a/b* tokens have a higher mean pitch than the *X* tokens, rendering them more salient.

All the tokens were spliced from the original recordings and concatenated into *aXb* strings with 250 ms within-string pauses between nonce words(similar to previous studies, [10–11,14,23]). Strings were approximately 2s in duration and were played separated by 750ms pauses.

### Participants

This study was approved by the Ethische Toetsings Commissie Linguïstiek (ETCL) of Utrecht University. Informed consent was obtained from all parents of infants tested, prior to testing.

Infants were recruited through written invitation to parents, whose addresses were provided by the Utrecht municipality. A total of 29 infants were included (15 females, 14 males), with an average age of 18 months and 16 days (range: 18 months and 4 days—19 months). Infants included had normal birthweight (2500–4500 grams), were not pre- or post-term (had a gestation period of 37–42 weeks), and had no known neurological, hearing or vision problems.

An additional 36 infants were tested but not included, due to: low birthweight (*n* = 3), less than 37 weeks gestation period (n = 2) oxygen shortage at birth (n = 1), vision impairment (n = 1), failure to retrieve information about birthweight /gestation period from parents (n = 2), fussiness, crying or fatigue (n = 18), completing fewer than 2 valid consistent or 2 valid inconsistent trials (n = 6), or technical error (n = 3). The drop-out rate due to infant behavior (fussiness, crying, etc.) is 28% (18/65), which is comparable to previous studies (32% in Kerkhoff et al. [23] and 29% in Gómez [10]). Exclusion due to short looking times (completing fewer than 2 valid trials of each type) is also similar to previous studies (6/65, 9% the same as in Kerkhoff et al. [23]).

Valid trials were trials where infants’ looking times were *at* least 2 seconds: we used this criterion similar to previous studies [10–11,23] because an *aXb* string was on average 2s in duration, and infants must hear at least one full string in a test trial in order to determine whether the final element *b*_*i*_ is predicted by the initial elements *a*_*i*_.

From the total of 232 test trials of the 29 infants included (8 test trials per infant), 31 were excluded for totaling a looking time shorter than 2 seconds (not enough time for the infant to perceive a full *a*_*i*_*Xb*_*i*_ string and identify the dependency). An additional 4 trials were excluded due to experimenter error (the trial was ended too soon), leaving 197 valid trials (84.9%) to be analyzed.

In addition to providing details about the infants’ medical history, parents also completed the Dutch version of the MacArthur-Bates Communicative Development Inventory (N-CDI) [24], to establish receptive and productive vocabulary size at 18 months. All parents provided written consent for their child’s participation in the study.

### Procedure

The Headturn Preference Procedure [25] was employed, similar to previous NAD-learning studies [10–11,23]. Infants were tested individually, while seated on their caretaker’s lap in a sound-attenuated booth. Throughout the experiment, caretakers listened to music over headphones, ensuring that they were unaware of the stimuli being played and could not influence infants’ behavior. The stimuli played over two speakers located on either side of the infant, at eye-level. Each speaker was positioned behind a red light, consisting of three concentric rows of LEDs that lit up sequentially, while a third, green light of the same description was positioned on the wall directly in front of the infant. A camera was mounted above the green light and recorded the looking behavior of the infant. An experimenter in the adjoining room viewed the camera feed on a monitor and controlled the lights in the cabin. All parents provided written consent that the video recordings could be used to analyze the data, and that the data could be used for publication.

#### Familiarization

The green light was used to capture the infant’s attention, and as soon as the infant’s gaze oriented towards it, it was extinguished and one of the red lights on the side began blinking. The sound began playing over both speakers when the infant oriented toward the red light. Looking time to the blinking side lights was measured from the moment the infant oriented towards the light until the moment s/he looked away for more than two seconds. When the infant looked away for more than two seconds, the side light was extinguished, and the green light in front was lit again. As soon as the infant oriented towards the green light the procedure was repeated. The successive use of one or the other red side-light was quasi-randomized (the same side-light was not employed more than twice in succession).

The familiarization language consisting of the 48 *aXb* strings (presented in random order) was played continuously over both speakers, irrespective of the infant’s looking behavior (similar to previous studies [10–11, 23]). This ensured that the familiarization phase had the same duration for all infants (two minutes) and was not prolonged by the infants’ failure to visually attend to the stimuli (leading to increased drop-out rates). Infants’ looking time (the total amount of looking time to the red side-lights during familiarization) was calculated, to obtain an approximate measure of each infant’s attention to the familiarization material, on the assumption that visual fixation is a proxy for auditory attention. If looking time at familiarization predicts discrimination performance, then auditory attention is crucial to learning.

#### Contingency phase

Following the familiarization phase, infants were presented with a short contingency training phase (the duration varied according to the looking times of the infants, but it never exceeded 30s). The green light was used to get the infant’s attention, and as soon as the infant oriented to it, one of the red lights started blinking. When the infant oriented to the red side-light, a 1s pure tone of 440Hz was played repeatedly (with 125ms pauses in between) from the speaker on the side of the light for as long as the child fixated the red light. When the child looked away for more than two seconds the sound stopped, and the green light came on again (whereas if the child looked away for less than two seconds, the trial continued uninterrupted). The same procedure was repeated one more time with the opposite side-light. This two-trial contingency phase was intended to train the infants on the contingency of light and sound on their looking behavior, thus preparing them for the procedure that would be used subsequently in the test phase and reducing potential noise in the first few test trials due to infants familiarizing themselves with this procedure. Contingency training has been used successfully in previous infant experiments [8,26] but has not been used in NAD-learning experiments before [10–11,23].

#### Test phase

The test phase consisted of 8 trials, 4 consistent with G1 and 4 consistent with G2. The order of presentation was quasi-randomized (no more than two trials of the same type, G1- or G2-consistent, could occur simultaneously). Whether the first trial was consistent or not to the familiarization grammar of the infant was counterbalanced over infants. Each test trial contained a maximum of 15 *aYb* strings (played consecutively with 750ms pauses in between), randomly sampled from the 6 *aYb* combinations (3 novel *Y*s combined with 2 dependencies) created for each grammar, G1 or G2.

The procedure was the same as in the contingency phase. For each trial, as soon as the green light captured the infant’s attention it faded, and a red side light started blinking. When the child oriented to the red light the stimuli started playing, until the child looked away for more than two seconds and the trial stopped. Children’s looking time to the red light was measured in each trial. In order to obtain more precise measurements of the looking times, all the video recordings were re-coded offline, using PsyCode (a Mac application provided to our research institute courtesy of Judit Gervain and Luca Bonatti). The experimenter viewed the recordings, identifying look or look-away points in the recording on a frame-by-frame basis, and calculated the looking times based on these. Data from 8 of the 31 infants (randomly selected) was re-coded by a second coder using a different software (UiL OTS Video Coding System). In spite of these differences looking times per infant per trial showed a high correlation between the two coders (Pearson’s *r* = .971) and inter-rater reliability was very high (with a Cronbach’s Alpha of .985).

### Data analysis

Previous studies [10–11,23] used Repeated Measures ANOVAs to analyze this type of data, averaging looking times for consistent and inconsistent stimuli across trials. However, because they average looking times across trials, RM ANOVAs do not take into consideration the variation within the different (consistent or inconsistent) trials that may arise within a subject. For example, a child may look longer to some trials of the same type than others, depending on whether these trials occur later or earlier in the test phase. RM ANOVA takes this variation to be equal to 0 and assumes there are only 2 observations available per infant (mean looking times to consistent and to inconsistent stimuli) where in fact there are (up to) 8. RM ANOVA is therefore not a sufficiently conservative test—it assumes the data is less noisy than it actually is.

Linear Mixed Models can take into consideration variation both at the level of the Subject (differences between participants) and Trial Number (looking times differences across trials). In addition, Linear Mixed Models are robust with respect to missing data, which is particularly suited to infant datasets where short trials are excluded. We therefore used a Linear Mixed Model with Looking Times at test (LT, per participant per test trial) as the (continuous) dependent variable and Subject as random intercept. We used Grammar (G1 or G2) to control for potential stimulus biases. We introduced Trial Number (1–8) as factor to control for differences between, for instance, earlier trials (where the infants may show longer looking times) and later trials (where infants looking times may decline). We also included (expressive/receptive) vocabulary scores (N-CDI, as a proxy for language development at 18 months) as covariate: we wanted to control for the possibility that children’s level of language development (specifically vocabulary development) would influence their looking behavior, in particular that children with higher vocabulary scores would show better discrimination of the test stimuli.

Because LTs (across trials and participants) followed a logarithmic-like distribution (with a high frequency of short LTs and a low frequency of longer LTs), we used a log-transformation of the dependent variable LT.

### Results

Table 1 shows the mean looking times (across trials and across infants) to test trials consistent or inconsistent with the grammar of exposure, as well as vocabulary scores and total looking time at familiarization.

**Table 1:** Results for Experiment 1: Average looking times (LTacross infants and trials; the range is averaged per infant across trials) to test trials consistent or inconsistent to the familiarization grammar, mean receptive and productive vocabulary as measured by the N-CDI, and average amount of looking time at familiarization.

	Mean (Standard Deviation)	Range
**LT Consistent**	M = 10.396 s (SD = 4.83)	3.72–24.27 s
	Median = 11.27 s	
**LT Inconsistent**	M = 11.77 s (SD = 7.34)	3.91–35.65 s
	Median = 9.76 s	
**Productive vocabulary**	Raw M = 76.7 words (SD = 66.12)	0–259 words
	Percentile M = 59.58 (SD = 28.53)	1st– 95th perc.
**Receptive vocabulary**	Raw M = 249.7 words (SD = 102.97)	29–440 words
	Perc. M = 64.5 (SD = 27.5)	1st– 95th perc.
**LT at Familiarization**	M = 61.917 s (SD = 15.17)	31.11–88.29 s

As can be seen, infants in our sample varied greatly, both in terms of language development (receptive and productive vocabulary) and in terms of their looking times at familiarization. The mean N-CDI percentile scores for receptive and productive vocabulary were around 60, comparable to those reported by Kerkhoff et al. [23].

Linear Mixed Models with Trial Type (Consistent or Inconsistent) and Grammar (G1, G2) as fixed factors and Trial Number as covariate were run using IBM SPSS Statistics 25. We started from a model with Subjects as random intercept and Trial Type (the variable of interest) as fixed factor and introduced each fixed factor/covariate (Grammar—G1, G2; Trial Number; Vocabulary Scores) separately in the model. We used a likelihood-ratio test to compare between models. Grammar did not significantly improve the model, but Trial Number did. There was a near-significant effect of Trial Number (*F* (1, 197) = 3.635, *p* = .058): infants’ looking times seemed to decline across trials (suggesting loss of attention due to fatigue), with shortest looking times for the last (8^th^) test trial. There was no significant effect of Trial Type, namely no significant difference in LTs to test trials Consistent vs. Inconsistent with the familiarization grammar (*F* (1, 197) = .632, *p* = .428). Introducing N-CDI scores of (receptive or productive) vocabulary as covariate (and its interaction with Trial Type) also did not significantly improve the model, suggesting that language development did not explain looking behavior.

Furthermore, given that Lany and Gómez [22] found a gender effect in the discrimination (and generalization) of NADs (between word and word-class) at 12 months (girls showed a robust learning effect while boys did not), we wanted to control for the possibility of a gender difference in our data too. Introducing Gender (and its interaction with Trial Type) did not significantly improve the model but introducing Total Looking Time at Familiarization (TLTfam) did, although TLTfam did not turn out to be a significant predictor (*F* (1, 189) = .310, *p* = .578). This suggests that attention during familiarization may account for some of the variance in the data, even though it does not cross the significance threshold.

We also checked whether the nature of the first test trial influenced behavior during the subsequent test trials. Gómez, Bootzin and Nadel [27] showed such an effect when infants napped between the familiarization and test phase of a NAD-learning experiment: the first test trial guided preference for consistent vs. inconsistent stimuli during the subsequent test trials [28]. The authors concluded that sleep promoted a more abstract type of learning: one where item-specific information was lost, but the learners retained the abstract notion of a relationship between non-adjacent elements. When refamiliarized with the language in the first test trial, infants used that abstract knowledge to identify the item-specific dependencies in trial 1.

In the current experiment, infants did not nap, although a contingency training phase did separate familiarization from test. However, if Gomez et al.’s [27] account is correct, naps should not be unique in determining infants to use the first test trial to guide their preference. Rather, in general, every time infants need to recover item-specific information to support learning (whether that information was lost or was never present at familiarization) they should use the first test trial to recover that information, and that first test trial could guide their subsequent preference.

**Fig 1:**
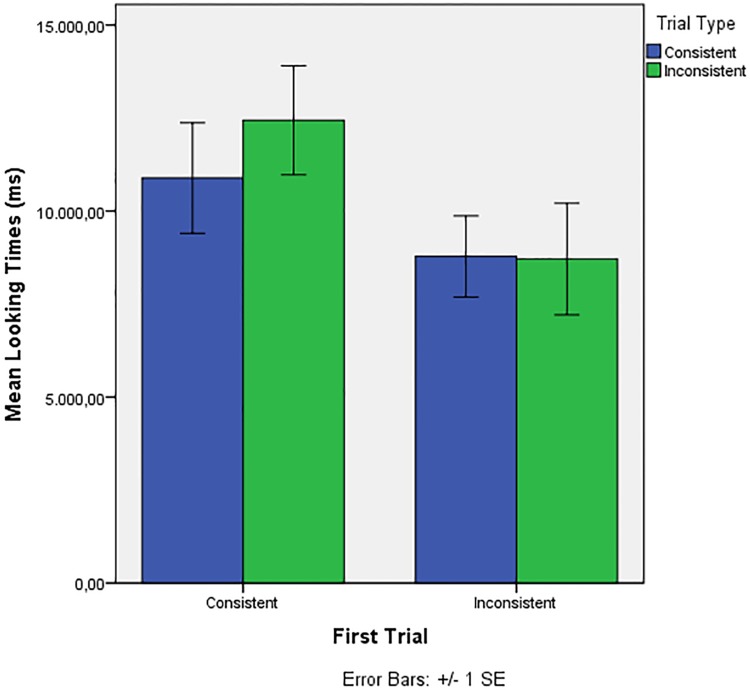
Results of Experiment 1. Average looking times to trials consistent or inconsistent with familiarization (Trial Type, trials 2–8) depending on the nature of the First Trial (Consistent/Inconsistent).

In the current experiment, because the stimuli at test were novel, the first test trial may be crucial in re-familiarizing the infants with the dependencies *in their novel contexts*. Therefore, we ran the Linear Mixed Model again, this time excluding LTs for the first trial from the data, and including First Trial (Consistent vs. Inconsistent) as a fixed factor. The best model included Trial Type and First Trial as fixed factors and Trial Number and TLTfam as covariate. This model showed a significant effect of First Trial (*F* (1, 163) = 4.257, *p* = .041), a near- significant effect of Trial Number (*F* (1, 163) = 3.003, *p* = .085) and no other effects. Infants who heard a first trial consistent with the familiarization (18 infants) subsequently showed longer looking times overall than infants who heard an inconsistent first trial (13 infants). Importantly, there was no significant interaction between First Trial and Trial Type (adding this interaction did not significantly improve the model, and when added, the interaction was not significant: *F* (1, 163) = .963, *p* = .328). In contrast to Gómez and colleagues’ findings [27–28], infants did not show a significant preference in trials 2–8 for the type of stimulus they heard during the first trial (see Fig 1).

Note that for all models run for this first Experiment, the final Hessian Matrix (based on the variances and covariances of the random effects) was not positive definite, prompting caution in interpreting the results. One possible reason is the introduction of subject as random intercept: the Hessian Matrix can only be positive definite if there is variation in intercepts between subjects (looking times vary between more than within subjects). It is possible that in the current data the random intercept was similar for all subject. We therefore ran all the analyses reported here excluding subject as a random intercept: the final Hessian Matrix was positive definite and the results did not differ from the ones reported here.

### Discussion

In Experiment 1 we set out to investigate infants’ ability to generalize NADs. Infants were exposed to 48 *aXb* strings, with two *a_b* dependencies and 24 different *X* items. Therefore, while they heard each of the dependencies they were meant to acquire 24 times, they only heard each of the 24 intervening *X* elements twice. According to previous research [10–11], this high variability and low frequency of the intervening material should be beneficial to the detection of *a_b* dependencies. Furthermore, previous findings show that 18-month-olds are capable of forming categories between sets of elements occurring in the same context, and of generalizing to novel elements [16,29]. We therefore predicted that the high variability and low frequency of the intervening *X* element should prompt young learners to develop a quasi-abstract *a_b* frame where any *X* element (of a quasi-abstract category *X*) can be inserted. To investigate that, we tested infants’ discrimination of novel *a*_*i*_*Yb*_*i*_ from *a*_*i*_*Yb*_*j*_ strings at test.

The results yielded no learning effect in the group of infants tested. In what follows we turn our attention to two potential reasons why this effect was not observed: (i) the possibility that the methodology employed in this experiment was not appropriate for capturing the predicted effect, or (ii) the possibility that generalization of NADs may still be limited at the age of 18 months.

The current study follows very closely previous research from two different laboratories [10–11,23]. The finding that 18-month-olds are capable of detecting NADs in simple *aXb* strings has therefore been replicated a number of times, including with Dutch infants. The methodology employed here is identical to the methodology of these previous studies (the same Headturn Preference Procedure, same selection criteria for the participants, comparable drop-out rates, etc.) in all but two specific aspects: the way the stimuli were recorded, and the introduction of a contingency training phase between the familiarization and the test phase.

The stimuli were recorded in the same way as Grama et al. [14]: while the *a/b* tokens were recorded in the same way as in the previous studies of Gómez [10–11] and Kerkhoff et al. [23], the *X* tokens were recorded in carrier sentences in the slot of direct object nouns. S1 Table shows the acoustic properties of the stimuli we used compared to the stimuli used in Kerkhoff et al. [23]. As can be seen, the stimuli were comparable in duration and intensity, but not in pitch—the stimuli in the current study were higher-pitched, particularly the *a/b* tokens. An important consequence of the way stimuli were recorded was that in the Kerkhoff et al. [23] study *X* items had lower mean pitch over their first syllable (despite being stress-initial words). Because the *aXb* strings were read out in a child-friendly voice, the reader emphasized the *a/b* words and de-accented the word in between. The *X* elements in the current study were recorded separately in contexts where they were in a position of focus/prosodic prominence (direct object nouns preceded by a prosodically reduced functional word). Their first syllable had higher mean pitch that the overall words.

There is no *a priori* reason to assume that the de-accented first syllable of the *X* token facilitated the extraction of the *a_b* dependency in the Kerkhoff et al. [23] study. In that study and the present one, *a/b* tokens still had higher mean pitch than the *X* tokens (first syllable or overall), and were thus highly salient. Furthermore, in Grama et al. [14] adult participants were exposed to stimuli recorded in exactly the same way as here and showed successful learning of the dependencies. These learners failed to learn the *aXb* language when the stimuli did not have a prosodically natural contour, but showed an improvement in learning as soon as the prosodic contour was amended. Thus, adults are very particular about the type of stimuli they can learn from—in particular with respect to prosodic contour—but they showed robust learning with stimuli similar to those used in the current experiment. This raises the question of why infants should not be able to learn from the same type of stimuli.

A second methodological difference between Experiment 1 and previous NAD-learning studies is the introduction of a contingency training phase before the test phase. It is unclear why this contingency phase should have any negative effect on learning. It is brief, containing only two trials, and is in fact intended to reduce the noise in the data by familiarizing infants with the procedure of the test trials. It has been used successfully with the Headturn Preference Procedure [8,26]. One potential disadvantage is that it prolongs the experiment, increasing the possibility of loss of attention towards the end of the experiment–which might be linked to the decline in looking behavior during the last trials of the test phase. However, the contingency phase never lasted longer than 30 seconds. Furthermore, if the contingency phase increased fatigue, we would expect it to also prompt higher drop-out rates due to fussiness or insufficient valid trials. Instead our drop-out rates were comparable to previous NAD-learning studies that did not use this contingency phase.

Finally, while we cannot eliminate the possibility that small methodological changes are responsible for the lack of a learning effect in Experiment 1, we must also consider the possibility that generalization may still be difficult for 18-month-olds, under the conditions of this experiment. As pointed out previously, by 18 months infants are capable of generalizing over classes of items. Aslin & Newport [30] proposed that this generalization may arise as a result of high variability and low item frequency: when the input presents high variability, learners will find it difficult to fully encode individual items; furthermore, when a variety of items occur interchangeably in the same context(s), these will be grouped together in a class, such that statistical computations can apply over classes of items. However, when items can be fully encoded because they are frequent in the input or have become sufficiently familiar to the learner, sensitivity to item-specific patterns can arise. In this experiment infants heard as many as 24 different *X* items, each of which was iterated only twice. It is difficult to believe that infants formed item-specific representations for each (or most) of the *X*s, so that they memorized specific *aXb* strings instead of abstracting the general *a_b* frame. Indeed, this strategy would run counter to Gómez and Maye’s [10–11] findings that children benefit from the high variability and low frequency of the intervening *X*.

An alternative explanation is that infants did not fail to learn the dependencies but rather failed to attend to them at test. Previous studies have shown that even when infants are familiar with non-adjacent dependencies, they may have a hard time recognizing them in certain contexts, especially where working memory demands are high [12]. One study in particular [31] claimed that these working-memory constraints may be modulated by the familiarity or processability of the intervening element: dependency-recognition can be blocked when the intervening material is not easily identifiable and quickly processed. Soderstrom and colleagues [32] also showed that 16-month-olds could track dependencies between functional morphemes over familiar words (i.e. discriminate *These* *chair**s*… from *These *chair_*…), or in passages with both familiar and unfamiliar intervening words (*These* *chair**s**…*, *These* *meep**s**…*) but not over unfamiliar/nonce words alone (*These* *meep**s* vs. *These *meep_*…). Thus, if the target dependency was found only in contexts where it straddled a nonce word, infants’ attention might have been captured by the novelty of the intervening nonce word, and they failed to discriminate the correct and incorrect dependencies (see also [21]).

A similar account could hold for the current experiment, if we consider that a difficulty to identify/process the intervening element can block recognition of dependencies [31–32]. Because the intervening *Y*s in the test phase were novel, infants may have taken longer to categorize (or process) them, or their attention may have been directed towards the novel *Y*s and away from the crucial *a_b* dependencies; because of these higher processing demands of the novel *Y* items, upon reaching the string-final *b* element learners might have already lost track of the string-initial *a* element, therefore failing to detect whether the non-adjacent dependency was in fact grammatical. Future research could address this confound by familiarizing infants with the novel *Y*s separately (i.e. not embedded in *aYb* strings). If infants are familiar with the *Y*s, but the *aYb* strings are still novel, then the generalization of *a_b* dependencies can be dissociated from the novelty of the individual *Y*s themselves.

## Experiment 2

### Introduction

As pointed out above, tracking morpho-syntactic dependencies in natural languages requires detecting NADs between functional morphemes that are not perceptually salient and generalizing those NADs to novel contexts. In Experiment 2 we investigate 18-month-olds’ ability to track dependencies between functional-like units. Grama et al. [14] showed that adult learners can successfully detect dependencies between prosodically non-salient units over intervening words that are prosodically more marked, as long as these dependencies are integrated into prosodically natural phrases.

This was particularly relevant because NAD-learning has been hypothesized to serve the acquisition of morpho-syntactic dependencies in natural languages, which are instantiated between prosodically reduced elements—functors (which are often prosodic clitics, or affixes), spanning lexical words with lexical stress and higher prosodic prominence. Functional and lexical morphemes differ in their prosodic properties [18], and this distinction is marked by a variety of perceptual cues [17,19, 20]; these cues can be employed by infants from the moment of birth to distinguish between the two categories [33]. Although infants familiarize themselves with most of the functional morphemes of their language by the end of their first year of [34–36], they also show a preference for listening to lexical over functional words [37–38], and an early inability to encode functors in full phonological detail [35–36, 39]) suggesting that their attention is captured by the more perceptually salient elements in their input.

The research question we address in this experiment is whether the specific perceptual cues to functional elements facilitate the detection of dependencies between them, or whether the lack of perceptual salience of these functors makes infants less likely to attend to the relationships between them. Just as Grama et al. [14] did with adults, we test the hypothesis that *Gestalt principles of similarity* are conducive to detecting NADs [13,40–41] in infants, whereby dependencies are more easily learned between elements that perceptually similar to each other but distinct from the environment. Adults can learn dependencies between both prosodically salient and prosodically reduced elements. At the age of 18 months, infants are already tracking morpho-syntactic dependencies between prosodically non-salient functional morphemes in natural languages [12, 31]. We predict that in an artificial grammar learning paradigm as well, 18-month-olds should be able to learn dependencies between functor-like nonce-words over lexical-like nonce-words, despite the lack of perceptual salience of the target elements.

Although Gómez & Maye [11] found that 18-month-olds tracking NADs in an artificial grammar showed a novelty preference, we cannot make a precise prediction about the direction of preference in this experiment Because our stimuli are less salient, the difficulty of the learning task may be greater causing a shift towards a familiarity preference ([11] and references cited therein). Simply observing a statistically significant difference between looking times to consistent vs. inconsistent stimuli is sufficient to constitute evidence of learning.

An alternative possibility is that learning is not reflected in an overall difference in looking times between consistent and inconsistent trials. Gómez, Bootzin and Nadel [27] showed that infants who napped between the familiarization and test phase showed a more abstract type of learning: they used the first test trial to guide their preference in the subsequent trials 2–8. Thus, if the first test trial was inconsistent with familiarization, then infants showed a preference for inconsistent trials, and if infants first heard a consistent trial the opposite pattern was observed. The authors concluded that napping before test allowed infants to obtain a more abstract representation of the *a_b* dependencies, where item-specific information (about which *a*_*i*_ corresponded to which *b*_*i*_) was lost, requiring the infants to use the first test trial to recover this information.

Although the infants in our study did not nap between familiarization and test, we might expect a similar effect to arise. Firstly, there is an intervening contingency training phase, which exposes infants to a different type of stimulus (pure tone). Secondly, functional-like morphemes are non-salient, and difficult for infants to fully phonologically encode [35–36,39]. With only a two-minute familiarization to the language, learners might develop weak memory representations for the non-salient *a/b* elements, which may decay across the intervening contingency training phase. Therefore, a pattern of results similar to Gómez, Bootzin and Nadel [27] is also likely to occur in our study, even without a nap between familiarization and test.

### Materials

The nonce words used in this experiment were identical to those in Experiment 1. The stimuli were created as follows: both *a* and *b* tokens were recorded in carrier sentences in the position of functional morphemes preceding or following a noun stem, within a noun-phrase in the direct-object slot of a Subject-Verb-Object sentence. Thus, while *a* was recorded in the slot of the determiner preceding the noun (2), *b* was recorded as the suffix on the noun stem (3) (in Dutch, there is a morpho-syntactic dependency between the diminutive inflection on the noun and the neutral determiner *het*).

(2) *Ik zie __ zebra'tje*.
  I see ___ zebra.DIM.
  e.g. *Ik zie* ***tep*** *zebratje*.
(3) *Ik zie het zebra__*.
  I see the zebra__.
  e.g. *Ik zie het zebra****lut***.

The *X* stimuli for Experiment 2 were the same as the ones employed for Experiment 1, and all the stimuli for Experiments 1 and 2 were recorded in the same session. Acoustic properties of the new *a* and *b* tokens, as analyzed using Praat 5303, are presented in S2 Table. The grammars G1 and G2 were constructed in the same way as for Experiment 1, except the pauses between the words in an *aXb* string were not 250 ms but 100 ms. Grama et al. [14] showed that learning dependencies between non-salient nonce-words is facilitated only with short 100ms inter-word pauses, just as in natural languages functional morphemes are prosodic clitics, and cannot be isolated by long pauses from the lexical stem they attach to. The test items were not novel *Y* stimuli from Experiment 1: instead of expecting infants to generalize to novel *aYb* strings, in the test phase we used strings taken from the familiarization phase. The three *X* items from the familiarization that were also used at test were *domo*, *kasi* and *wadim*. As in Experiment 1, the 48 familiarization strings were played in random order with 750ms pauses in between. The familiarization phase lasted 1 minute and 40 seconds (shorter than in Experiment since the *a/b* tokens themselves were shorter in duration).

### Participants

This study was approved by the Ethische Toetsings Commissie Linguïstiek (ETCL) of Utrecht University. Informed consent was obtained from all parents of infants tested, prior to testing.

Infants who did not participate in Experiment 1 were recruited in the same way as before. A total of 39 infants were included (21 females, 18 males), with an average age of 18 months and 15 days (range: 18 months and 2 days– 19 months). Infants included had normal birthweight (2500–4500 grams), were not pre- or post-term (had a gestation period of 37–42 weeks), and had no known neurological, hearing or vision problems.

An additional 35 infants were tested but not included due to: low birthweight (*n* = 1), gestation period of less than 37 weeks (n = 1), failure to retrieve information about birthweight and gestation period from parents (n = 3), fussiness, restlessness, crying or fatigue (n = 19), completing fewer than 2 valid consistent or 2 valid inconsistent trials (n = 2), parental interference or presence of distraction (n = 4) or technical error (n = 6). Again, drop-out due to fussiness was comparable to previous studies (19/74, 25.7%), and exclusion due to completing fewer than 2 valid trials of each type was very low (2/74, 2.7%).

From a total of 312 test trials of the 39 infants included (8 test trials per infant), 44 were excluded because for totaling a looking time shorter than 2 seconds. An additional 6 trials were excluded due to parental interference (n = 1), fussiness (n = 1), and experimenter error (n = 4), leaving 262 valid trials (83.97%).

Parents again completed the Dutch version of the MacArthur-Bates Communicative Development Inventory (N-CDI) [24], to establish receptive and productive vocabulary size at 18 months.

### Procedure

The procedure was identical in all respects to that of Experiment 1. Data from 9 of the 40 infants (randomly selected) was re-coded by a second coder. Looking times per infant per trial showed a high correlation between the two coders (Pearson’s *r* = .958) and inter-rater reliability was very high (with a Cronbach’s Alpha of .978).

### Results

As can be seen in Table 2 infants again varied greatly, both in terms of language development (receptive and productive vocabulary) and in terms of looking times at familiarization. The looking time at familiarization in this experiment is shorter than Experiment 1 because the familiarization itself was shorter (1 minute and 40s instead of 2 minutes). In both Experiment 1 and 2 infants oriented to the blinking sidelights about 50% of the time at familiarization.

**Table 2:** Results of Experiment 2: Mean looking times (across trials and infants) to test trials consistent or inconsistent with familiarization, mean N-CDI scores (raw and percentiles), and the total looking time at familiarization.

	Mean (Standard Deviation)	Range
**LT Consistent**	M = 10.724 s (SD = 5.923)	3.41–26.63 s
	Median = 9.76 s	
**LT Inconsistent**	M = 10.754 s (SD = 5.449)	4.05–25.84 s
	Median = 9.43 s	
**Productive vocabulary**	M Raw = 54.06 words (SD = 40.45)	10–158 words
	M Percentile = 54.09 (SD = 20.32)	15th– 90th perc.
**Receptive vocabulary**	Raw M = 229.52 words (SD = 106.02)	72–473 words
	Perc. M = 55. 88 (SD = 28.5)	10th– 99th perc.
**LT at Familiarization**	M = 50.363 s (SD = 16.20)	24.86–93.4 s

We used a Linear Mixed Model with (a logarithmic transformation of) LT as dependent variable, Subject and as random intercept and Trial Type as fixed factor, and introduced the other factors (Grammar, Trial Number, Vocabulary Scores) one by one in the same way as before. The best model included Trial Number as covariate and Trial Type as fixed factor, and only yielded a significant effect of Trial Number (*F* (1, 228.02) = 9.283, *p* = .003) and no significant effect of Trial Type (*F* (1, 228.827) = .628 *p* = .429). Thus looking times to Consistent and Inconsistent Trials did not differ significantly; however, children showed a decline of looking times across the test phase again, with shortest looking times during the last (8^th^) trial. Adding Gender did not significantly improve the model, but adding Total Looking Time at Familiarization did: this time, TLTfam was a significant predictor (*F* (1, 38.299) = 5.464, *p* = .025), suggesting that infants who showed longer looking times during familiarization also showed overall longer looking times at test.

As in the first experiment, we considered the possibility that infants may have been taking the first test trial as a cue for looking behavior across the subsequent trials [27]. Although infants did not nap between familiarization and test, the may have ‘lost’ item-specific representations of the relevant *a_b* dependencies in another way. Previous literature has suggested that the low perceptual salience of functor-like elements prevents young infants from forming fully phonologically specified representations of these elements [35–36,39]. In the current experiment, infants were exposed for only 2 minutes with *aXb* strings where the *a* and *b* elements were functor-like, and therefore less perceptually salient. In addition, a contingency training phase intervened between familiarization and test, allowing item-specific representations of the non-salient *a/b*s to further decay. Therefore, it is possible that infants arrived at the test phase with insufficient item-specific representations of the target elements, and used the first test trial to recover that item-specific information necessary to track specific dependencies between *a* and *b* items.

**Fig 2:**
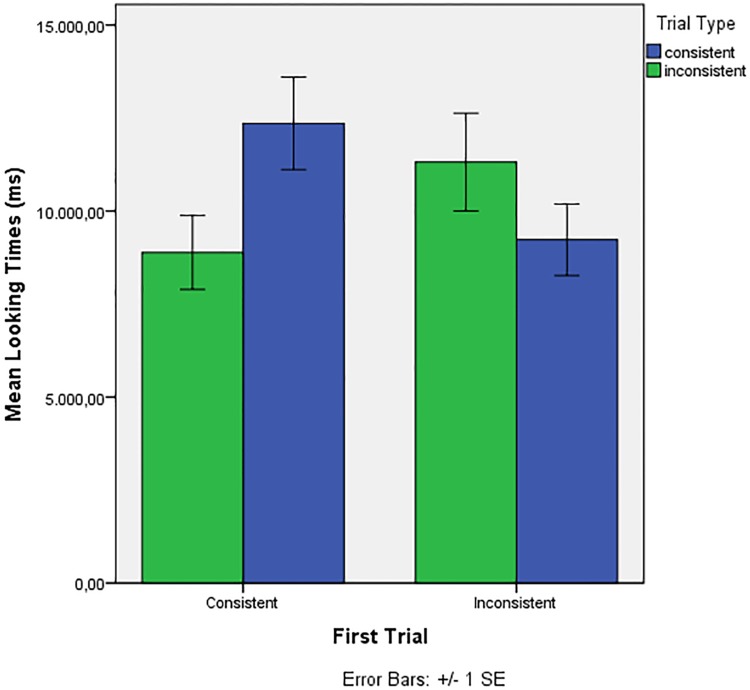
Results of Experiment 2. Average looking times to trials consistent or inconsistent with familiarization (Trial Type, trials 2–8) depending on the nature of the First Trial (Consistent/Inconsistent).

We excluded the first trial and reanalyzed the data introducing First Trial (Consistent, Inconsistent) as a factor. The best model included First Trial, Trial Type and their interaction. There was a significant interaction of First Trial* Trial Type (*F* (1, 189.187) = 8.147, *p* = .005), and no significant main effect of First Trial (*F* (1, 36.39) = .308, *p* = .582) or Trial Type (*F* (1, 189.187) = 1.838, *p* = .177). Fig 2 illustrates the First Trial* Trial Type interaction: infants who heard a first test trial consistent with the familiarization showed a preference for consistent stimuli in the subsequent test trials, while infants who heard and inconsistent test trial first showed a subsequent preference for inconsistent stimuli (see Fig 2).

Similar to Gómez, Bootzin and Nadel [27], infants seemed to develop a preference for the stimulus type they heard during the first test trial, and maintained that preference throughout the following test trials. Out of the 20 infants who heard an inconsistent first test trial, 12 subsequently showed longer looking times for inconsistent strings; out of the 19 who heard a consistent test trial first, 15 showed longer looking times for consistent strings. Therefore, 27 out of 39 infants showed a preference, in trials 2–8, for the dependencies they heard in the first test trial.

We created a new variable, Consistency with First Trial, which coded each of Trials 2–8 for whether they were consistent with the first trial. We reanalysed the data with First Trial and Consistency with First Trial as fixed factors. If, across the dataset and irrespective of the nature of the first trial, children showed a preference for dependencies consistent with the first trial, then we predicted only a significant effect of Consistency with First Trial. If, on the contrary, children exhibited different looking behaviour depending on the nature of the first trial, then we would expect a First Trial * Consistency with First Trial interaction. Neither First Trial nor the First Trial * Consistency with First Trial interaction significantly improved the Linear Mixed Model, and neither did Trial Number. The only factor that significantly improved the model was Consistency with First Trial, indicating a significant preference across trials 2–8 for items consistent with the first trial (*F* (1, 189.231) = 7.871, *p* = .006). Adding Total Looking Time at Familiarization again improved the model fit, and TLTfam was a significant predictor (*F* (1, 36.723) = 5.048, *p* = .031).

### Discussion

In this experiment we asked whether infants can detect NADs between prosodically distinct but reduced elements over prosodically more salient intervening material. Like in Experiment 1, we found no overall significant preference for one type of test item over another, but upon closer scrutiny the data revealed an interesting pattern.

However, a closer look at the data showed that looking preference depended significantly on the nature of the first test trial: during test trials 2–8, infants showed a significant preference for the dependencies that were consistent with what they had heard during the 1^st^ trial. This in itself is evidence that infants could discriminate test trials based on the crucial difference under investigation: the specific *a*_*i*_ to *b*_*i*_ mapping in the *a*_*i*_*Xb*_*i*_ strings.

The finding that infants use the first test trial to guide their preference for the rest of the test trials is not new. Gómez, Bootzin and Nadel [27] found a similar effect in a NAD-learning experiment where infants napped between the familiarization phase and the test. They familiarized 15-month-olds with an *aXb* language for 15 minutes in their home and then tested them on recognition of the *a_b* dependencies in familiar *aXb* strings 4 hours later. Infants who did not nap (or napped for under 30 minutes) between familiarization and test exhibited a significant preference for the correct dependencies, as predicted [11]. However, infants who napped (for more than 30 minutes) did not show an overall preference for one type of stimulus or the other. Instead they showed a significant preference for the type of stimuli that they heard in the first test trial. Furthermore, infants who napped but were exposed to an *aXb* language with low variability of the *X* elements (3, which was shown not to support NAD-learning [10–11]), showed neither pattern, suggesting that simply hearing the first test trial did not induce a preference in the subsequent test trials.

The authors concluded that when infants learned at familiarization and napped subsequently, sleep erased the item-specific memory traces of the *a*_*i*_*_b*_*i*_ mappings (e.g. *tep* goes with *lut* and *sot* goes with *jik*). Gómez and Edgin [42] attribute this to the fact that sleep before 18 months does not seem to support (item-specific) memory consolidation (which develops between 18–24 months), but rather seems to facilitate generalization (abstracting the knowledge that a dependency exists, without specifying the dependent elements). Thus the abstract representation of a dependency between the first and last word in an *aXb* string could have been retained and activated in the first test trial, where infants could use it to identify the specific *a*_*i*_*_b*_*i*_ mappings from just a short amount of exposure.

There are crucial differences between the study of Gómez and colleagues [26] study and Experiment 2. Experiment 2 presented 18-month-old (not 15-month-old) infants with a shorter exposure (only 2 instead of 15 minutes) to a language with perceptually non-salient *a/b* tokens, had a much shorter break between familiarization and test (a contingency training phase of no more than 30s, but where infants continued to receive stimulation in the form of tones) and did not involve sleep.

Nevertheless, we propose that Gómez and colleagues’ [27] interpretation of their results could be extended to the current findings as well. Infants in this experiment were presented for a short period of time to a language where the target words instantiating the regularity to be learned were prosodically ‘reduced’. As pointed out before, infants between 6 and 12 months of age have difficulties encoding functional-sounding words or morphemes [35–36,39]. Although after their first birthday infants become sensitive to the phonological detail of many of the functors in their language (presumably due to prolonged exposure), it is not unlikely that they retain a difficulty to phonologically encode prosodically reduced elements from only brief exposure to them. It is furthermore not surprising that the fragile phonological representations obtained from this brief exposure should deteriorate over an intervening contingency training phase, where infants are required to attend to a different type of stimuli.

One alternative explanation for the current results may be that infants did not retain the abstract notion of an *a*_*i*_*_b*_*i*_ dependency from familiarization at all; instead, it is possible that they simply memorized full *a*_*i*_*Xb*_*i*_ chunks from the first test trial and recognized them subsequently in the next test trials. Although we cannot eliminate this possibility, we would expect that if children were able to quickly memorize *a*_*i*_*Xb*_*i*_ chunks from the first test trial, then they would also show this ability in Experiment 1, thus circumventing the need to generalize dependencies from *a*_*i*_*Xb*_*i*_ to *a*_*i*_*Yb*_*i*_ strings. We found no evidence for this. In Hupbach et al. [28] children who did not nap within a few hours after exposure also showed no evidence of their discrimination of test trials 2–8 being guided by test trial 1 (or indeed of overall discrimination of test trials). Thus, children learn from the first test trial only under specific circumstances—most likely, that is, when the familiarization leaves them with mental representations that they can match to the test items.

To conclude, even without a sleep episode between familiarization and test, infants in the current experiment may have lost the item-specific representations of *a*_*i*_*_b*_*i*_ dependencies. Subsequently, they used the first test trial to recover these representations and re-establish the correct dependencies. This interpretation crucially rests on the assumption that 18-month-olds commit fragile or incomplete representations of prosodically reduced (functor-like) elements to memory, and that these memory traces are easily deteriorated by subsequent exposure to something else, in this case, to a pure tone and the association between looking behavior and sound/visual stimulus.

## General discussion

The ability to detect NADs in spoken input has been attested with infants as young as 15 months old [11], and has been hypothesized to contribute to the acquisition (starting from around 18 months [12]) of morpho-syntactic dependencies in natural languages.

If this hypothesis is true then NAD-learning should provide infants with knowledge of dependencies that can be generalized to novel context, since morpho-syntactic dependencies themselves are generalizable to different context (e.g. *The princess* *is* *kiss**ing* *the frog*, vs. *The general* *is* *ber**ating* *his army*). The first research question we tackled was: (i) Can infants generalize NADs to novel contexts?. Secondly, NAD-learning should provide infants with the ability to track dependencies between perceptually non-salient units in the input, as functional morphemes in natural languages can often be prosodically ‘reduced’. Therefore the second research question we pursued was: (ii) Can infants detect NADs between prosodically non-salient elements? Because adults proved capable of both tasks, we predicted that infants would show significant learning in both cases.

Experiment 1 showed no evidence of generalization of NADs to novel contexts. We considered the possibility that something in the methodology, stimuli or particular group of participants in this experiment might have led to a null result, but found it less likely, since NAD-learning has been replicated with a variety of stimuli, in different labs, with both adult and infant groups. The introduction of a ‘contingency phase’ in between familiarity and test was new in our design, but this methodology has been employed before successfully [8,26] in an experimental setup quite similar to our own (with a Headturn Preference procedure, between the familiarization and test phase). Furthermore, Experiment 2 showed evidence of learning even while employing this same contingency phase.

We suggested an alternative possibility: that novel Y items directed attention and processing resources away from the *a_b* dependencies. Soderstrom et al. [32] showed that even with real morpho-syntactic rules, children could only track non-adjacent dependencies if at least some of the intervening elements were familiar. This account may run into the following potential problem: if novelty in the intervening element guides attention away from the frequently recurring *a*_*i*_ and *b*_*i*_ elements, then being exposed to *a*_*i*_*Xb*_*i*_ strings with highly variable (and infrequent) *X*s should inhibit learning, because with every new string the intervening *X* would most often be novel. Furthermore, under Gómez’s [11] learning conditions children are not likely to be memorizing the individual *X*s at all, to be able to detect whether they are novel or familiar. Rather, the proposal is that children might form an abstract category *X* defined by its position in the string. It is the formation of this abstract category, and the inability to encode the individual *X*s in memory, which might prompt infants to find a better source of information in the stable *a*_*i*_*_b*_*i*_ dependencies, prompting NAD-learning.

To address this objection, it is important to consider the structure of the familiarization and test phases: while familiarization exhibited the strong variability of the *X* elements, with only 2 repetitions for each of the 24 *X*s, at test children heard 3 different *Y*s repeated for as long as they gazed at the visual stimuli, over 8 test trials. As a separate learning phase, therefore, the test phase offered less variability and often more repetition of the *Y* elements. Therefore, whereas at familiarization children might have abstracted the *a*_*i*_*_b*_*i*_ dependencies across highly variable *X*s, at test they could use their knowledge of these dependencies to make inferences about (/as frequent frames to help categorize) the new *Y*-elements they were hearing. Thus, while maintaining all the assumptions about NAD-learning stated above, our results could be explained by the fact that while familiarization prompted generalization, this generalization was not possible at test where the lower variability of items prompted more item-specific representations.

Whether or not this interpretation based on attention is on the right track, our results are consistent with Soderstrom et al.’s [32] findings in suggesting that children around the ages of 16–18 months might not yet have a fully mature ability to generalize NADs. At least a partial familiarity with the intervening material might promote NAD-learning at this age (at least in the early stages of learning). Preliminary findings with adults in our lab have also tentatively suggested a possible advantage (in generalizing *a*_*i*_*_b*_*i*_ dependencies) for learners who actually show higher post-test familiarity with the *X*s (presented at the familiarization) than with the *a*_*i*_ and *b*_*i*_ elements. We are currently tailoring our methodology to more accurately capturing the relationship between NAD-learning and item-specific retention of *a*_*i*_, *b*_*i*_ or *X* elements. The extent to which NAD-learning benefits from some item-specific knowledge of the intervening material remains an open question. Future research could test children’s generalization to *Y* elements that are familiar from different contexts but have never been straddled by a dependency—eliminating the novelty confound while preserving the task of generalization.

As opposed to Experiment 1, Experiment 2 showed a learning effect that could be linked to generalization of a different type. Infants exposed to an *a*_*i*_*Xb*_*i*_ language with prosodically ‘reduced’ *a/b* tokens (but more salient *X* items) employed the first test trial in a subsequent test phase to guide their discrimination of correct *a*_*i*_*Xb*_*i*_ vs. incorrect *a*_*i*_*Xb*_*j*_ strings. We proposed that infants could detect NADs between prosodically reduced elements, although the memory traces of these item-specific *a*_*i*_*_b*_*i*_ one-to-one mappings might deteriorate over time without sufficient repetition. In other words, children may have detected a highly abstract *a_b* frame where the specific *a*_*i*_*_b*_*i*_ pairings where underspecified.

We have seen from previous literature that infant representations of perceptually non-salient, functor-like items are fragile and often under-specified [34–39]. But if functors are so non-salient for infants compared to lexical units, then tracking patterns between them in the input might be expected to be difficult too. What facilitated infants in our Experiment 2 to track these dependencies at test? One explanation is the purported reliance of NAD-learning on Gestalt principles of perceptual similarity [13]: although functors are perceptually non-salient, and often surrounded by more salient lexical elements, relationships between them might be tracked more easily because they are perceptually similar to each other and distinct from the environment.

An alternative explanation is that both 18-month-olds (Experiment 2) and adults [14] already have linguistic experience that suggests to them that frequent, non-salient elements may be functional in nature, and therefore often involved in important structural rules. By 18 months infants have become familiarized with the functional elements in their language [34–36] and their combinatorial properties [43–46], including morpho-syntactic dependencies [12,3,47–49]. This linguistic experience might bias learners of 18 months to pay special attention to functor-like elements in the input, since these functors have often proved to be informative. Thus, learners may track dependencies between non-salient elements not because of Gestalt principles of perception, but rather because they are primed to do so by their prior linguistic experience.

Irrespective of which account turns out to be correct, the results of Experiment 2 suggest that 18-month-olds can keep track of dependencies between functor-like elements over lexical-like elements. This learning ability may prove highly useful in tracking morpho-syntactic dependencies in natural languages. And even when item-specific representations may be erased quickly, infants seem to maintain some type of abstract information about the existence of a dependency, which later exposure can capitalize on to rapidly re-familiarize learners with the specific dependencies. Future research is called upon to identify whether, with multiple presentation across multiple sessions (better emulating the input infants receive during language acquisition), stronger and more item-specific representations of NADs can be committed to long-term memory.

To conclude, we have put forth evidence suggesting that 18-month-olds may be equipped with a NAD-learning mechanism that might prove relevant to language acquisition. These learners may derive abstract representations of NADs by the age of 18 months, but their ability to generalize these dependencies to novel contexts may not have fully matured by this age. The relationship between item-specific and non-item-specific representations at this age is complex and interesting, and decidedly merits further investigation.

## Supporting information

S1 Table Stimuli for Experiment 1. Acoustic properties of the stimuli used in Experiment 1 compared to Kerkhoff et al. (2013); *X* elements were also used in Experiment 2.
(DOCX)

S2 Table Stimuli for Experiment 2. Acoustic properties of the a/b stimuli.
(DOCX)

S1 File Data from Experiments 1 and 2 and Inter-rater reliability. Looking times per infant per trial, along with participant information—gender, CDI scores, Total Looking Time at Familiarization for Experiments 1–2, and the inter-rater reliability for a random subsample of each experimental group.
(XLSX)

S2 File Protocol. The experimental protocol for Experiments 1 and 2.
(DOC)
