# Supplementary material for: Learning and generalizing non-adjacent dependencies in 18-month-olds: A mechanism for language acquisition?
Source: PLoS One. 2018 Oct 11;13(10):e0204481. doi: 10.1371/journal.pone.0204481 (PMC6181290; doi:10.1371/journal.pone.0204481)
Supplement: S1 Table — Acoustic properties of the stimuli used in Experiment 1 compared to Kerkhoff et al. (2013); X elements were also used in Experiment 2. (DOCX) [file pone.0204481.s001.docx]

**S1 Table**

| Experiment 1 | | | | | Kerkhoff et al. (2013) | | | |
| --- | --- | --- | --- | --- | --- | --- | --- | --- |
| Items | Duration  (ms) | Intensity  (dB) | Pitch  (Hz) | Pitch 1^st^ syll. | Duration  (ms) | Intensity  (dB) | Pitch  (Hz) | Pitch 1^st^ syll. |
| *a* | | | | | | | | |
| Tep | 320 | 59.37 | 384.9 | - | 357 | 59.93 | 284.7 | - |
| Sot | 498 | 57.44 | 375.9 | - | 484 | 61.66 | 300.6 | - |
| *b* | | | | | | | | |
| Lut | 443 | 59.32 | 378.8 | - | 523 | 60.41 | 306.3 | - |
| Jik | 429 | 57.81 | 362.1 | - | 423 | 60.01 | 292.3 | - |
| *X* | | | | | | | | |
| wadim | 572 | 61.15 | 260 | 300.7 | 596 | 62.4 | 204.7 | 178.9 |
| kasi | 510 | 61.77 | 243.5 | 279.3 | 486 | 60.53 | 201.5 | 182.9 |
| poemer | 415 | 63.49 | 267.7 | 361.7 | 497 | 63.08 | 204.6 | 204.9 |
| kengel | 413 | 65.8 | 297.7 | 355.9 | 511 | 62.46 | 215.6 | 208.6 |
| domo | 516 | 64.41 | 257.9 | 315.4 | 584 | 64.57 | 211.4 | 190 |
| loga | 573 | 62.29 | 255.7 | 314.8 | 604 | 63.27 | 229.2 | 204.8 |
| gopem | 593 | 62.2 | 249.1 | 285.8 | 697 | 62.58 | 226.8 | 200.8 |
| naspu | 591 | 62.51 | 276.1 | 323.1 | 625 | 62.62 | 228.4 | 193.5 |
| hiftam | 603 | 61.26 | 246.4 | 317.5 | 645 | 62.04 | 229.9 | 241.6 |
| dieta | 402 | 59.05 | 251.2 | 362.6 | 595 | 59.37 | 198.6 | 191.6 |
| vami | 578 | 63.15 | 245.5 | 303.5 |  |  |  |  |
| snigger | 572 | 58.53 | 233.9 | 353.5 |  |  |  |  |
| rogges | 541 | 59.34 | 288.7 | 335.8 |  |  |  |  |
| densim | 601 | 58.1 | 270 | 393.9 |  |  |  |  |
| fidang | 607 | 59.36 | 253.5 | 392.4 |  |  |  |  |
| rajee | 598 | 60.53 | 244.2 | 355.2 |  |  |  |  |
| seta | 626 | 59.22 | 211.5 | 262 |  |  |  |  |
| noeba | 524 | 63.28 | 248.6 | 292.3 |  |  |  |  |
| plizet | 591 | 61.18 | 245.6 | 356.1 |  |  |  |  |
| banip | 594 | 60.95 | 263.5 | 306.2 |  |  |  |  |
| movig | 641 | 62.48 | 240.5 | 293.2 |  |  |  |  |
| sulep | 563 | 61.27 | 254 | 333.5 |  |  |  |  |
| nilbo | 652 | 60.92 | 231.3 | 274.7 |  |  |  |  |
| wiffel | 456 | 59.68 | 226.7 | 361.4 |  |  |  |  |
| *X’* | | | | | | | | |
| klepin | 576 | 59.39 | 259.1 | 346.9 | - | - | - | - |
| lotup | 600 | 60.02 | 293.6 | 336.3 | - | - | - | - |
| tarsin | 617 | 61.38 | 244.2 | 299.5 | - | - | - | - |
| Mean (X) | 561 | 59.27 | 254 | 326 | 584 | 62.29 | 215 | 200 |
