# Supplementary material for: Learning and generalizing non-adjacent dependencies in 18-month-olds: A mechanism for language acquisition?
Source: PLoS One. 2018 Oct 11;13(10):e0204481. doi: 10.1371/journal.pone.0204481 (PMC6181290; doi:10.1371/journal.pone.0204481)
Supplement: S2 Table — Acoustic properties of the a/b stimuli. (DOCX) [file pone.0204481.s002.docx]

**S2 Table**

| Item | | Duration (ms) | Pitch (Hz) | Intensity (db) |
| --- | --- | --- | --- | --- |
| a | tep | 302 | 247.1 | 53.52 |
|  | sot | 445 | 254.4 | 56.41 |
| b | lut | 346 | 183.4 | 46.41 |
|  | jik | 345 | 185.2 | 44.67 |
