# Supplementary material for: Learning and generalizing non-adjacent dependencies in 18-month-olds: A mechanism for language acquisition?
Source: PLoS One. 2018 Oct 11;13(10):e0204481. doi: 10.1371/journal.pone.0204481 (PMC6181290; doi:10.1371/journal.pone.0204481)
Supplement: S2 File — The experimental protocol for Experiments 1 and 2. (DOC) [file pone.0204481.s004.doc]

**PROCOTOL – NON-ADJACENT DEPENDENCY LEARNING**

**UiL-OTS Babylab**

**Before testing**

The **caller** contacts the parents and makes an appointment. They ask the parents to fill in the **ANAMNESIS** and **N-CDI** and send it; as soon as an appointment is made s/he fills in the **participant sheet** and leaves it on the table.

Make sure there are copies of the **consent form** available.

**Setting up the hardware**

Turn on the computers, the speakers in the testing booth, and connect the video feed to the desktop in the experimenter’s cabin.

Start the experiment script.

Walk **carefully** behind the white panels (**don’t upset wires!! If you do call technician immediately!**) to place the **green light** in the **front**; make sure it is placed centrally, fasten it with the screw on the little shelf behind, and cover it with the white cover that only leaves a circular hole for the light

Dry-run the experiment before the parents show up, to **check that the speakers are working** and that the experiment is running smoothly

Check that the **music** is playing in the parents’ headphones and that the video is recording.

Fill in the participants’ details in the particiant sheet.

**When the parents+babies come in**

Parents wait in the main entrance hallway to be picked up; tell them a bit about the experiment if the child wants to play with the toys in the room; otherwise bring to testing cabin immediately.

Ask the parents to sign the **consent form** agreeing to allow their child to participate in the experiment, and allowing us to save and analyze the video data. Explain that all the data (video, Anamnesis, N-CDI questionnaire) is confidential.

**Instruct the parents about the procedure**: keep the child on your lap facing the front light and have the headphones on during the entire experiment; do not influence the child in any way, do not point to the lights, re-orient them only when the **green** light is on, **do not talk** [tell them the experiment presents blinking lights in order to monitor the child’s attention, and that they will have masking music over headphones]; if the baby is fussy/crying/uncomfortable, the experiment can just be stopped at any time

**START THE VIDEO RECORDING** by pressing start on the little recorder window.

S**tart the experiment**  by pressing the start button on the experiment window, have **headphones** on to hear what goes on in the cabin; communicate with the parents through the headphone speaker.

S**ignal look/lookaway** by pressing the **purple** and, respectively, **red** button on the **buttonbox.**

If the child is **fussy/crying** ask the parents whether they want to stop, and if so abort the experiment **Ctrl+F4**.

**When the experiment is over**

Stop & save the **videofile** in the experiment folder with id of the participant.

Give the parents the **gift for the baby** (books in the cupboard with the label **18**) and **travel reimbursement** if necessary (reimbursement money is in the box in the gifts cupboard, have them **sign** the sign-up sheet in the same box and put it back)

Ask the parents whether they’ve sent in **ANAMNESIS** and **NCDI** – if they haven’t done it yet, tell them to please do it; ask the parents if they can be approached for **future** **research** or not and write it on the participant sheet

When the parents have left, fill in the rest of the participant sheet with details of the testing, with the **reasons for exclusion** if it’s the case, then punch holes in the form and put it in the **experiment folder.**

Check that the results have been saved.
